# Mirror-image discrimination in the literate brain: a causal role for the left occpitotemporal cortex

**DOI:** 10.3389/fpsyg.2014.00478

**Published:** 2014-05-21

**Authors:** Kimihiro Nakamura, Michiru Makuuchi, Yasoichi Nakajima

**Affiliations:** ^1^Human Brain Research Center, Graduate School of Medicine, Kyoto UniversityKyoto, Japan; ^2^National Rehabilitation Center for Persons with DisabilitiesTokorozawa, Japan

**Keywords:** mirror-image discrimination, transcranial magnetic stimulation, visual orientation invariance, occipitotemporal cortex, visual word-form area

## Abstract

Previous studies show that the primate and human visual system automatically generates a common and invariant representation from a visual object image and its mirror reflection. For humans, however, this mirror-image generalization seems to be partially suppressed through literacy acquisition, since literate adults have greater difficulty in recognizing mirror images of letters than those of other visual objects. At the neural level, such category-specific effect on mirror-image processing has been associated with the left occpitotemporal cortex (L-OTC), but it remains unclear whether the apparent “inhibition” on mirror letters is mediated by suppressing mirror-image representations covertly generated from normal letter stimuli. Using transcranial magnetic stimulation (TMS), we examined how transient disruption of the L-OTC affects mirror-image recognition during a same-different judgment task, while varying the semantic category (letters and non-letter objects), identity (same or different), and orientation (same or mirror-reversed) of the first and second stimuli. We found that magnetic stimulation of the L-OTC produced a significant delay in mirror-image recognition for letter-strings but not for other objects. By contrast, this category specific impact was not observed when TMS was applied to other control sites, including the right homologous area and vertex. These results thus demonstrate a causal link between the L-OTC and mirror-image discrimination in literate people. We further suggest that left-right sensitivity for letters is not achieved by a local inhibitory mechanism in the L-OTC but probably relies on the inter-regional coupling with other orientation-sensitive occipito-parietal regions.

## Introduction

The human and primate ventral visual system is known to spontaneously generate a common and invariant representation from a visual object image and its mirror reflection, irrespective of their left-right orientation (Eger et al., 2004; Vuilleumier et al., 2005; Dehaene et al., 2010b; Freiwald and Tsao, 2010). For humans, this mirror-image generalization probably relies on a fast neural process occurring at ~200 ms after stimulus onset (Eddy and Holcomb, 2009), but seems to be partially “suppressed” through literacy acquisition. That is, literate adults are known to have greater difficulty in recognizing mirror images of letters than those of other objects, whereas this is not the case for illiterate people (Kolinsky et al., 2011; Pegado et al., 2014). Recent functional magnetic resonance imaging (fMRI) data show that such category-specific sensitivity in mirror-image processing relies on the left visual word-form area (VWFA) in the left fusiform gyrus (Dehaene et al., 2010a,b; Pegado et al., 2011).

However, it remains unclear how the strong behavioral sensitivity to letter/word orientation is achieved in this and adjacent left occpitotemporal cortex (L-OTC). More specifically, while this region is thought to represent abstract shape-invariant identities of letters (see Dehaene et al., 2005 for review, and see also Rothlein and Rapp, 2014), it is unknown whether the same region comprises a local inhibitory circuit for suppressing mirror-image generalization only for letters and words. More specifically, it is possible that mirror-image representations are covertly generated even from letter stimuli and then suppressed via a local feedback circuit in the L-OTC. This is expected because (1) a recent event-related study has shown that early neural responses to masked letters/words (i.e., ~250 ms after stimulus onset) do not differ between normal-oriented and mirror-reversed stimuli (Dunabeitia et al., 2011), and (2) such lateral inhibition of non-canonical inputs seems to reflect an ubiquitous feature of the neural mechanism involved in early sensory processing (Srinivasan et al., 1982) and play a role in shaping response tuning of higher-order sensory pathways (Carandini and Heeger, 2012). Indeed, human extrastriate cortex may comprise a lateral inhibition mechanism in which neuronal populations responsive to one stimulus category suppress those responsive to another category (Allison et al., 2002). It is therefore plausible that a category-specific inhibitory circuit for mirror-reversed letters develops within the L-OTC through literacy training.

On the other hand, it is also possible that the L-OTC comprises no such orientation-sensitive inhibitory mechanism for mirror-image discrimination. This is because this region *per se* has been associated with abstract, shape- and orientation-invariant representations of visual stimuli (Dehaene et al., 2005) and thus might be unable to differentiate their left-right orientations. If this is the case, mirror-image discrimination during visual word perception may not occur inside the L-OTC, but rather rely on input signals from other orientation-sensitive regions, such as the lateral occipital cortex (LOC) (Eger et al., 2004; Vuilleumier et al., 2005; Dilks et al., 2011) and posterior parietal cortex (PPC) involved in spatial recognition (Poldrack and Gabrieli, 2001).

**Figure 1:**
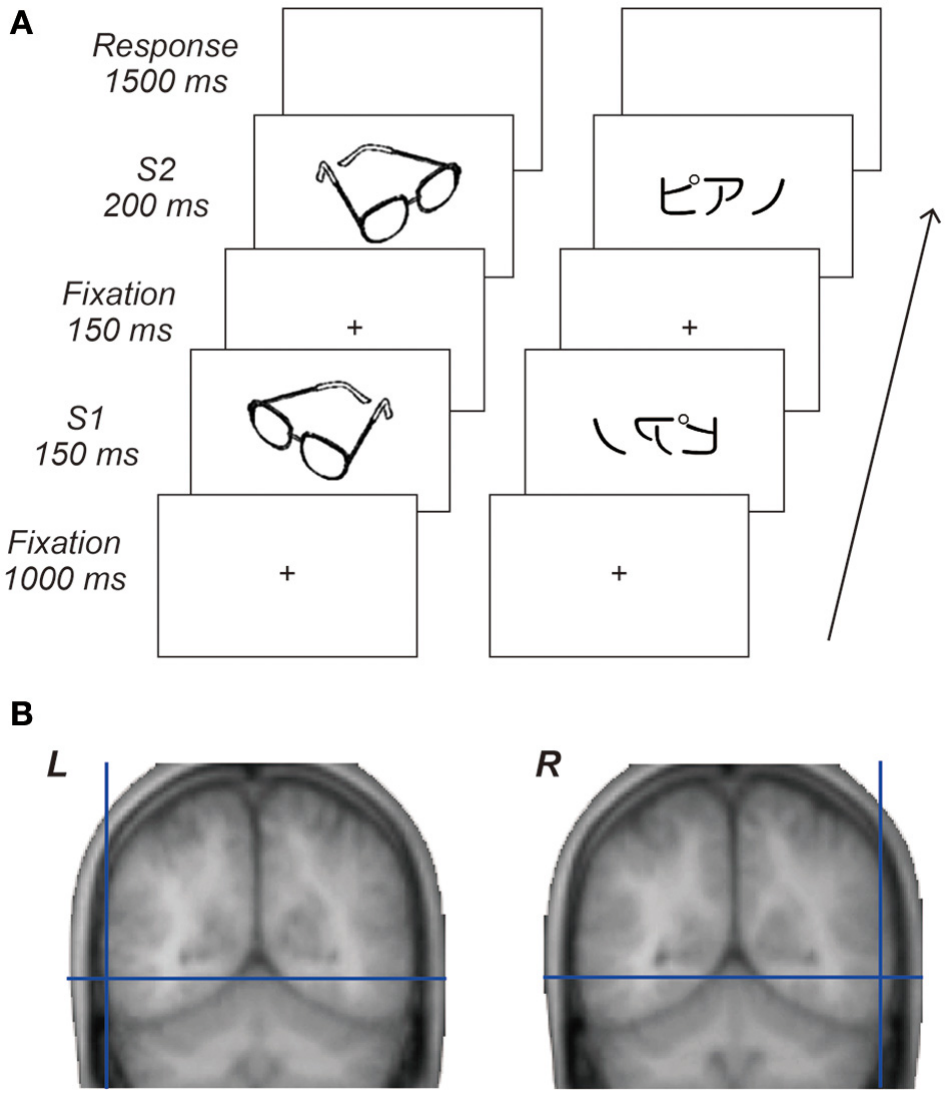
**Behavioral task and cortical target regions**. **(A)** Sequence of visual stimuli. Each trial comprised central fixation, a first stimulus (S1), central fixation, a second stimulus (S2) followed by a response period. Visual stimuli for S1 and S2 were either identical or different images taken from the same category and presented either in the same or mirror reversed orientation. Participants responded by key-press as quickly and accurately as possible to decide whether or not paired stimuli were identical regardless of their orientation. **(B)** Locations of the cortical target structures in the occipitotemporal areas. The average coordinates of these cortical targets across participants were *x* = −62, *y* = −60, *z* = −5 for the L-OTC and *x* = 61, *y* = −57, *z* = −5 for the R-OTC.

In the present study, we examined these two possibilities by applying transcranial magnetic stimulation (TMS) to the left and right OTC during a same-different judgment task (Figure 1A). We measured a behavioral impact of TMS on mirror-image recognition by varying the semantic category (letters and non-letter objects), identity (same or different) and orientation (same or mirror-reversed) of the first and second stimuli. Crucially, the two different models described above should predict different patterns of TMS-induced interference during mirror-image processing. On one hand, if the L-OTC comprises a category-specific inhibitory circuit for left-right discrimination, the visual recognition of mirror-reversed words should be facilitated when this region is disrupted by TMS. That is, a transient reduction of inhibitory signals is likely to accelerate the otherwise suppressed mirror-image processing for letter-strings, since mirror generalization is known to occur in both hemispheres (Eger et al., 2004; Vuilleumier et al., 2005; Freiwald and Tsao, 2010). On the other hand, if such local inhibition is not operating in the L-OTC, no behavioral facilitation should occur during mirror-image recognition when TMS is applied to this region. Rather, magnetic stimulation of the region would disrupt the orientation-invariant representations of stimulus identity, and thereby induce a delay in same-different judgment about mirror images. These effects should be strictly category-specific, i.e., detectable only for word stimuli and not for other visual objects.

## Materials and methods

### Participants

Twelve right-handed Japanese speakers participated in the present TMS experiment (age range 20–38 years, six females). All of them gave written informed consent prior to the TMS experiment. We additionally recruited a separate group of 18 Japanese participants (age range 19–45 years, seven females) for a control experiment without TMS (see Results). The protocol of this study was approved by the institutional ethical committee at the National Rehabilitation Center for Persons with Disabilities.

### Materials and procedures

Visual stimuli consisted of 48 Japanese words written in a syllabic script (katakana) and 96 black-and white drawings of objects (e.g., animals, clothes, faces, tools). Since printed words and other drawings greatly differ in physical features, it is possible that they also depart from each other in the degree of asymmetry. We therefore assessed the degree of asymmetry for the present stimuli using a pixel-based analysis. That is, visual images were binarized to remove white background pixels and then edge-detected using the Matlab image processing toolbox (Mathworks, USA), For each item, we determined the number of overlapping pixels shared by the filtered image and its left-right reversal, and computed the ratio of overlap against the whole filtered image. This analysis revealed that mean percentage overlap (*SD*) was 11.6 (5.7)% for words and 9.52 (3.99)% for objects, respectively, and did not differ from each other (*p* > 0.2, Wilcoxon rank-sum test). In addition, faces tended to be slightly more symmetrical than other non-face objects (10.1 (3.8) and 8.9 (4.1)%, respectively), but this difference neither reached significance (*p* = 0.18). These results thus confirmed no significant difference in the degree of asymmetry between words and other objects.

Each trial comprised central fixation, a first stimulus (S1), central fixation, a second stimulus (S2) followed by a response period (Figure 1A). Visual stimuli for S1 and S2 were either identical or different images taken from the same category and presented either in the same or mirror reversed orientation. Participants responded by key-press as quickly and accurately as possible to decide whether or not paired stimuli were identical regardless of their orientation (thus they should make a “same” response when S2 was a mirror image of S1). Each participant received two sessions of 240 randomly ordered trials. The order of the stimulation site was counterbalanced across participants. The experiment was therefore arranged in a 2 × 2 × 2 × 2 factorial design, treating S1–S2 stimulus identity (same vs. different), orientation (same vs. mirror-flipped), category (words vs. objects), and magnetic stimulation site (L-OTC vs. R-OTC) as within-participant factors. In addition, we performed a third 240-trial session in nine of the 12 participants to assess a non-specific, global impact of TMS by applying the same level of magnetic pulse to a distant control region, i.e., the vertex (Vx, see Results).

### TMS procedures

A high-resolution anatomical MRI was obtained for each participant prior to the TMS experiment. We selected the left and right OTC as target structures to assess the regional specific effects of TMS on mirror-image recognition. For the L-OTC stimulation, we targeted a posterior part of the left inferolateral temporal region ~25 mm posterior to the lateral edge of the transverse temporal gyrus, which overlaps the a subpart of the L-OTC known as the VWFA (Dehaene et al., 2005). On each participant's MRI, a right homologous region was identified as a target structure in the R-OTC. The average coordinates of these cortical targets across participants were *x* = −62, *y* = −60, *z* = −5 for the L-OTC and *x* = 61, *y* = −57, *z* = −5 for the R-OTC (Figure 1B) according to the standardized brain space defined by the Montreal Neurological Institute. In addition, the Vx was selected as an active cortical control site for each participant.

A single-pulse TMS was generated using two MagStim 200 magnetic stimulators connected to a 70 mm figure-of eight coil through a BiStim module (Magstim, UK). The coil was kept tangential to the skull for stimulating the OTC and Vx with the handle pointing backward parallel to the midline. TMS pulse was applied 100 ms prior to the onset of S2 at an intensity of 60% of the stimulator power output, which corresponded to 80~120% of the motor threshold of resting hand muscles. A single magnetic pulse at this stimulus intensity is estimated to suppress the local neuronal activity for approximately 100 ~ 200 ms (Moliadze et al., 2003). Using a 3D-navigation system (Nexstim, Finland), we tracked the position and orientation of the coil relative to the head at the rate of ~20 Hz to minimize their mutual displacement during the TMS session using our standard TMS procedures (see Nakamura et al., 2006, 2010).

## Results

### Effects of TMS on the left and right occipitotemporal region

**Figure 2:**
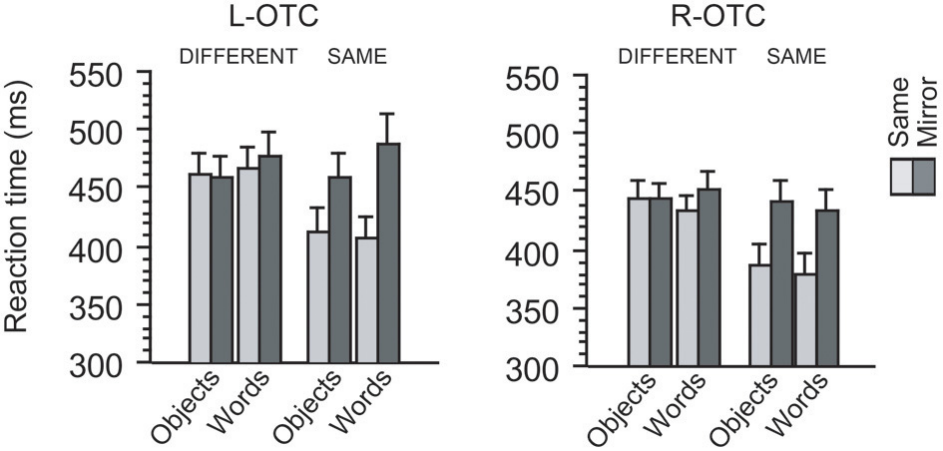
**Behavioral effects induced by the TMS of the left and right OTC**. For each TMS site, mean reaction times during same-different judgment are illustrated with respect to the identity, category, and orientation of visual stimuli. For each site, participants responded similarly when S1 and S2 differed in identity from each other (i.e., “different” trials) irrespective of their category and orientation. In same-identity trials, however, participants responded more slowly when S1 and S2 were mirror images than when they were identical. Moreover, this mirror recognition cost was greater for words than for objects when TMS was applied to the L-OTC, whereas no such category-specific effect emerged when TMS was applied to the R-OTC.

Participants made only few errors during the same-different judgment task [mean error rate (*SD*) = 2.81 (1.91)%]. We assessed reaction time data for correct responses (Figure 2) using 2 × 2 × 2 × 2 ANOVA treating site (L-OTC vs. R-OTC), category (words vs. objects), orientation (same vs. mirror-reversed) and identity (same vs. different) as within-participant factors (outliers > 3 *SD* above the mean were excluded from this and all subsequent analyses). First, overall latency did not differ between words and objects (*F* < 1). However, participants responded 28 ms more slowly in L-OTC stimulation than in R-OTC stimulation, whereas this left-right difference in TMS was significant (*p* = 0.003). These effects interacted with each other (*p* < 0.02), suggesting that the left-right asymmetry in TMS effects was greater for words (35 ms) than for objects (20 ms).

On the other hand, participants responded 33 ms more slowly in mirror trials than in same trials. This effect of orientation was highly significant (*p* < 0.001), but interacted with the effect of category (*p* < 0.02), suggesting that the behavioral cost of mirror recognition was greater for words (41 ms) than for objects (25 ms). Furthermore, the main effect of identity was also significant (*p* = 0.004) and interacted with that of orientation (*p* < 0.001), suggesting a net component of cognitive processing cost for recognizing mirror images as being identical. Indeed, the effect-size of identity was much greater when paired stimuli were in the same orientation (56 ms) than in mirror-flipped orientation (3 ms). This finding was expected because the orientation difference between S1 and S2 should yield a recognition cost in making “same” responses only when the stimuli are mirror images, whereas the orientation of stimuli is not important in making “different” responses when S1 and S2 are totally different images (we therefore performed further analysis restricted to same identity trials, as described below). These effects of identity and orientation produced no triple interaction, either with site (*p* > 0.1) or with category (*F* < 1), but showed a significant quadruple interaction with site and category (*p* = 0.04). This last finding suggests that the recognition cost for assimilating mirror images increases for words relative to objects when TMS was applied to the L-OTC (see below). Other interactions were all non-significant (*p* > 0.1).

We then assessed the effects of site, category, and orientation by restricting the analysis to “same identity” trials. This analysis revealed significant effects of site (*p* = 0.001) and orientation (*p* < 0.001) but not that of category (*F* < 1). Participants responded to objects 10 ms faster than to words in L-OTC stimulation, whereas this trend was reversed in R-OTC stimulation (i.e., ~7 ms faster to words than to objects), resulting in a significant cross-over interaction between site and category (*p* = 0.01). Response latency to objects was 51 ms slower in mirror trials than in same-orientation trials, whereas this “mirror recognition cost” (Pegado et al., 2014) was even greater for words (68 ms). Indeed, the effect of category on mirror recognition cost was marginally significant (*p* = 0.06). More importantly, there was a significant triple interaction between site, category and orientation (*p* = 0.01), suggesting that the between-category difference in mirror recognition cost was enhanced by the disruption of the L-OTC relative to that of the R-OTC.

### Effects of TMS on the vertex

We then performed a third session with 240 trials in which TMS was delivered to a distant control region (Vx). The behavioral paradigm and TMS procedure were the same as those in the main experiment. This control experiment is required because magnetic stimulation of the R-OTC might change the activation level of the L-OTC via callosal connections between the left and right hemispheres. That is, neuropsychological and neuroimaging data show that these homotopic regions may exert a mutually inhibitory influence on each other (Forss et al., 1999; Fink et al., 2000; Ueki et al., 2006; Koch et al., 2008; Nakamura et al., 2012b).

**Figure 3:**
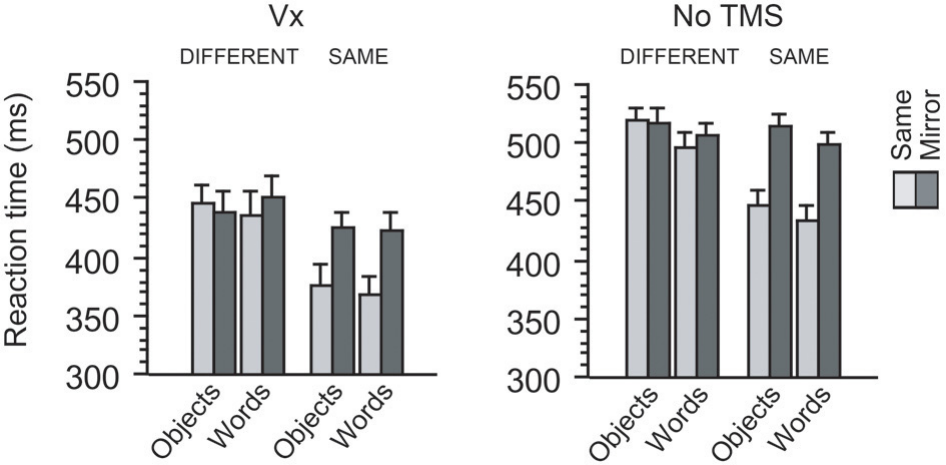
**Behavioral results in two control experiments**. When TMS was applied to a control site (Vx) distant from occipitotemporal regions, participants showed the similar amount of mirror recognition cost between words and objects. This pattern of behavioral cost during mirror image processing was also observed when no TMS was applied during the same behavioral task. Thus, the effects of category, orientation, and identity overall produced the similar patterns of impact on reaction times between the two experiments (see Results).

Again, participants made few errors during the same-different judgment task [mean error rate (*SD*) = 4.52 (4.92)%]. Reaction time data for correct responses are presented in Figure 3. Since our main interest was to compare the behavioral effects of Vx stimulation with those of L-OTC and R-OTC, we ran a 2 × 2 × 2 × 2 ANOVA separately for each of the left and right OTC, treating site (OTC vs. vertex), category (words vs. objects), orientation (same vs. mirror-reversed), and identity (same vs. different) as within-participant factors. Therefore the critical comparison here is the main effect of site and its interaction with other factors.

First, the R-OTC vs. Vx comparison revealed that the main effect of site never approached significance (422 vs. 420 ms, *p* > 0.5). Moreover, this effect did not interact with any other factors (*p* > 0.2 for all interactions). Thus, these findings suggest that the behavioral effects induced by Vx stimulation did not differ significantly from those induced by R-OTC stimulation.

Next, the L-OTC vs. Vx comparison revealed that overall latency was slower in L-OTC stimulation (442 ms) than in Vx stimulation (420 ms), although this ~22 ms difference did not reach significance (*p* = 0.20). The effect of identity was significant (*p* = 0.003) and produced a trend of interaction with the effect of site (*p* = 0.1). The effect of site interacted neither with that of orientation nor with that of category (*p* > 0.2 for both). These four factors (site, category, orientation, and identity) produced no significant triple interactions (*p* > 0.2 for all). Lastly, however, there was a significant quadruple interaction (*p* = 0.01), similarly to the comparison between L-OTC and R-OTC in the main experiment (see above). Thus, these results additionally support the previous finding that L-OTC stimulation produces a regional specific impact on the mirror recognition process.

### Comparisons with a non-TMS baseline

**Figure 4:**
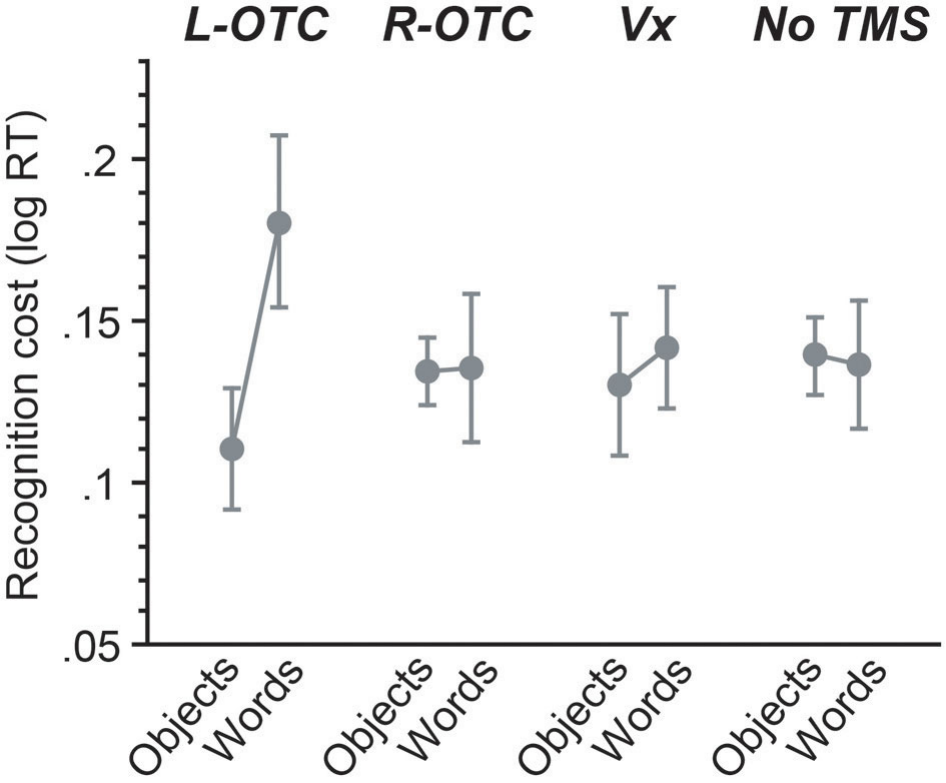
**Across-session comparisons for mirror recognition cost**. For each session, the magnitude of mirror recognition cost was calculated by subtracting log-transformed reaction times for mirror trials from those for same-orientation trials. Magnetic stimulation of the L-OTC produced a large impact on mirror-image recognition for words but not objects, whereas all other sessions showed the similar pattern of behavioral effects without significant between-category differences (see Results).

We further conducted a behavioral experiment without TMS with a separate group of 18 participants to determine the baseline pattern of mirror-image recognition during the same-different judgment task. These participants also made few errors during the same-different judgment task [mean error rate (*SD*) = 2.69 (1.88)%]. On the other hand, overall responses were >50 ms slower in this non-TMS experiment compared to the TMS experiment (see Figure 3). Probably, this large between-group difference should be attributed to some non-specific behavioral facilitation effects of TMS, known as “inter-sensory facilitation” (e.g., Terao et al., 1997). We therefore transformed reaction time data into a logarithmic scale to compare the mirror recognition cost between different sessions. The behavioral index for mirror recognition cost (Figure 4) was obtained by selecting only same-identity trials and then calculating log RT differences between same orientation trials and mirror trials for each category for each session.

For this mirror recognition cost, we then examined the effect of TMS and its interactions with other factors by contrasting each of the three TMS sites with the non-TMS control. First, the L-OTC vs. non-TMS comparison revealed no significant effect of TMS on mirror recognition cost (*F* < 1). However, there was a marginally significant effect of category (*p* = 0.05), suggesting that the magnitude of mirror-recognition cost was greater for words than for objects. Importantly, there was a significant interaction between TMS and category (*p* = 0.03), suggesting that the category-specific impact on recognition cost was greater for L-OTC relative to the non-TMS. On the other hand, both the R-OTC vs. non-TMS and the Vx vs. non-TMS comparisons revealed that the effects of TMS and category and their interaction were all non-significant (*p* > 0.5 for all). These findings suggest that the overall pattern of mirror recognition cost did not differ between R-OTC, Vx, and non-TMS sessions.

## Discussion

Recent brain imaging studies suggest that fluent reading rests on a distributed bilateral network extending from the lateral frontal region to ventral and dorsal visual areas (Dehaene et al., 2005; Cohen et al., 2008; Nakamura et al., 2012a). Since written language is a recent cultural invention dating back only ~5000 years, this extensive reading network should be shaped by imposing learning-related plastic changes upon evolutionarily older neural systems as a function of cognitive processing demands of reading (see e.g., Szwed et al., 2014). In particular, mirror-image discrimination is likely to rely on such experience-dependent process occurring in the higher-order visual system during literacy development. That is, whereas the human ventral visual area involved in object recognition is generally known to represent visual objects and their mirror reversals as being the same (Eger et al., 2004; Vuilleumier et al., 2005; Dehaene et al., 2010b), the intrinsic propensity for mirror-image generalization should be partially suppressed through literacy training, since many writing systems include minimal pairs of mirror-image letters, such as “b” vs. “d” and “p” vs.”q” (Dehaene et al., 2005). Literacy development is indeed likely to involve such unlearning process, because visual sensitivity to left-right orientation has been shown to increase with literacy acquisition (Kolinsky et al., 2011; Dunabeitia et al., 2013). At the neural level, the mirror-image discrimination during reading has been associated with a subpart of the L-OTC termed the VWFA (Dehaene et al., 2010a,b; Pegado et al., 2011).

In the present study, we examined whether or not the VWFA system previously associated with mirror-image discrimination comprises a local inhibitory mechanism for suppressing neural activations induced by mirror-reversed letter-strings. We observed that magnetic stimulation of the L-OTC interfered with mirror-image recognition more greatly for words than for other objects. In contrast, the transient disruption of the R-OTC did not produce such category-specific impact on mirror-image processing. Rather, additional analyses of control experiments showed that the main effects of category and orientation during R-OTC stimulation did not differ in effect-size from those obtained from the Vx and no-TMS sessions, suggesting that TMS of the R-OTC did not interfere with mirror-image recognition. These findings therefore suggest that the observed increase in mirror processing cost for words is a regional specific effect of L-OTC stimulation, which is distinct from the effects observed for other control sites, including R-OTC and Vx.

The present results further suggest that the L-OTC in itself does not exert inhibitory influence over mirror-image representations of letter-strings, because the visual processing of mirror words should be facilitated when such local inhibitory circuit is disrupted by the magnetic stimulation of the L-OTC. Rather, our results revealed that TMS of this region produced a significant delay in mirror-image recognition only for words and not for objects. Since the same-different judgment of visual stimuli and their mirror reversals relies on their shared, orientation-invariant representations, this finding concurs with the notion that the same part of the ventral visual system stores such higher-order, invariant identity of letter-strings (Dehaene et al., 2005). It is therefore likely that mirror-image discrimination during skilled reading does not occur inside the VWFA but rather involve other orientation-sensitive cortical regions, such as LOC (Eger et al., 2004; Vuilleumier et al., 2005) and PPC (Poldrack and Gabrieli, 2001).

Indeed, the left and right LOCs are thought to constitute a “posterior letter recognition system” involved in the visual analysis of letter shapes (Tarkiainen et al., 2002; Ellis et al., 2009). It seems rather plausible that literacy training develops a feed-forward mechanism favoring normally oriented words over their mirror images, since visual face recognition, i.e., another well-known example of expert visual recognition, is thought to rely on a strong structural and functional coupling between these extrastriate regions and OTCs that is at least partially enhanced by visual experience (Fairhall and Ishai, 2007; Gschwind et al., 2012). If this is the case, mirror-image discrimination may be achieved in the VWFA by collecting strong bottom-up activations of orientation-sensitive LOC neurons produced by normally oriented letters and filtering out weaker activations produced by mirror-reversed letters. Indeed, recent neurobiological data show that stimulus selectivity, at least for early visual cortex, is mediated by such feed-forward mechanism incorporating non-linear properties of cortical neurons (e.g., spike threshold, contrast saturation), rather than by classical lateral inhibition circuits (Priebe and Ferster, 2008). Thus, if the higher-order ventral visual area also relies on the similar feed-forward connections, the strong selectivity of the VWFA to normal oriented letters/words as observed in previous fMRI studies (Dehaene et al., 2010a; Pegado et al., 2011) may arise from a bottom-up activation of abstract orthographic codes driven by excitatory signals from the earlier, orientation-sensitive LOC regions.

On the other hand, mirror-image discrimination for letters may also partially rely on the dorsal visual pathway, including the PPC, which is generally known to be sensitive to the orientation of visual stimuli (Culham and Valyear, 2006) and modulate the activation level of the object-sensitive extrastriate areas in the control of spatial attention (Serences et al., 2004; Shomstein and Behrmann, 2006). Recent brain imaging data indeed suggest that the left and right PPCs participate in a tightly interconnected network for reading across different writing systems (Cohen et al., 2008; Nakamura et al., 2012a). Importantly, however, neuropsychological data suggest that damage to the PPC causes left-right disorientation for non-linguistic objects but not for letters (Davidoff and Warrington, 2001; Priftis et al., 2003; Vinckier et al., 2006). It is therefore possible that efficient mirror-image discrimination during reading is mediated by the ventral visual area independently of the parieto-occipital region (see Pegado et al., 2011 for further discussion). Even if this is the case, however, it is still open whether and to what extent mirror-image discrimination of letters can occur automatically without focused attention. Rather, it might rely on top-down allocation of spatial attention, since, for instance, mirror-image letters (e.g., “b” and “d”) are more easily confused in peripheral vision than in central vision (Chung, 2010). Moreover, even mirror-image generalization, i.e., a more innate and intrinsic property of the ventral visual system and probably less attention-dependent process, seems to depend on spatial attention and does not occur automatically for unattended or unconsciously perceived visual stimuli (Bar and Biederman, 1998; Eger et al., 2004). Clearly, further behavioral and brain imaging data should be collected to determine the relative contribution of the dorsal attention-control system in mirror-image discrimination during expert visual word recognition.

To summarize, we found that mirror processing cost increased for written words and not for other objects when TMS was applied to the L-OTC. This finding suggests that this region *per se* does not comprise a local inhibitory circuit for suppressing mirror-image representations of letter-strings and better fits with a hierarchical model whereby the VWFA represents abstract identity of letter-strings by collecting feed-forward signals from earlier orientation-sensitive extrastriate regions (Dehaene et al., 2005). In addition, at the methodological side, an important advantage of TMS over other brain imaging techniques (e.g., fMRI, magnetoencephalography) is that it allows causal inferences about brain structure and function (Pascual-Leone et al., 1999). The present results hence provide new causal evidence showing that the L-OTC is specifically involved in mirror-image discrimination during fluent reading. Such visual expertise for letters would rely on a fine tuning of the ventral visual system through literacy development and thus represent a detectable behavioral-level signature of the literate brain.

### Conflict of interest statement

The authors declare that the research was conducted in the absence of any commercial or financial relationships that could be construed as a potential conflict of interest.
